# Supplementary material for: Colorimetric determination of urea using diacetyl monoxime with strong acids
Source: PLoS One. 2021 Nov 8;16(11):e0259760. doi: 10.1371/journal.pone.0259760 (PMC8575183; doi:10.1371/journal.pone.0259760)
Supplement: S1 File — (PDF) [file pone.0259760.s001.pdf]

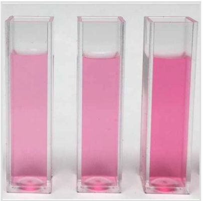

Colorimetric determination of urea V.3

noah.langenfeld [1], Laurenpayne [1], Bruce Bugbee[1]

[1]Crop Physiology Laboratory, Utah State University

1 Works for me Share

Version 3

Oct 07, 2021

dx.doi.org/10.17504/protocols.io.byvipw4e

USU Crop Physiology Laboratory

noah.langenfeld

ABSTRACT

This protocol measures the absorbance of urea in solution in complexation with diacetyl monoxime at 520 nm and is linearly proportional to concentration up to 5 mM urea.

DOI

dx.doi.org/10.17504/protocols.io.byvipw4e

PROTOCOL CITATION

noah.langenfeld , Laurenpayne , Bruce Bugbee 2021. Colorimetric determination of urea. **protocols.io**
https://dx.doi.org/10.17504/protocols.io.byvipw4e
Version created by noah.langenfeld

WHAT'S NEW

Added purity of reagents.

KEYWORDS

urea, urea assay, colorimetric urea, urea test, thiosemicarbazide, diacetyl monoxime

LICENSE

This is an open access protocol distributed under the terms of the Creative Commons Attribution License, which permits unrestricted use, distribution, and reproduction in any medium, provided the original author and source are credited

CREATED

Oct 07, 2021

LAST MODIFIED

Oct 07, 2021

PROTOCOL INTEGER ID

53898

MATERIALS TEXT

thiosemicarbazide, diacetyl monoxime, water, sulfuric acid, phosphoric acid, ferric chloride

SAFETY WARNINGS

This protocol utilizes strong acids. Ensure proper precautions and safety equipment are used when dealing with these chemicals.

BEFORE STARTING

Ensure a lab coat, lab goggles, and plastic gloves are worn throughout this assay.

Mixed Acid Reagent Preparation

1

Dissolve **2.5 mg** ferric chloride (97% purity) in **45 mL** deionized water in a 250 mL volumetric flask.

2 Add **80 µl** of [M] **14.8 Molarity (M)** concentrated phosphoric acid (85 wt. % phosphoric acid in water).

3

> Caution: Preparation of [M] **4.7 Molarity (M)** sulfuric acid is highly exothermic. Slowly add acid to **100 mL** water and stir frequently to avoid spattering.

Prepare [M] **4.7 Molarity (M)** sulfuric acid by diluting **65.25 mL** concentrated sulfuric acid ([M] **18 Molarity (M)** , 98% purity) up to **250 mL** with deionized water.

4 Dilute ferric chloride, deionized water, and phosphoric acid mixture to **250 mL** with [M] **18 Molarity (M)** sulfuric acid.

5 Mix until dissolved.

Mixed Color Reagent Preparation

6 Add **20.9 mg** diacetyl monoxime (99% purity) and **52.4 mg** thiosemicarbazide (99% purity) to a 250 mL volumetric flask.

7 Dilute to volume (**250 mL**) with deionized water.

8 Mix until dissolved.

Urea Assay 10m 5s

9 Prepare known concentrations of urea at 0, 1, 2, 3, 4, and 5 mM for a calibration curve.

10 Fill a 600 mL beaker with **200 mL** deionized water.

11 Place the 600 mL beaker on hot plate and bring to a boil.

12 To test a sample, aliquot **1 mL** into a 20 mL glass test tube.

13 Add **2 mL** Mixed Acid Reagent.

14 Add **2 mL** Mixed Color Reagent.

15 Ensure contents of test tube are vigorously mixed by vortexing for at least **00:00:05** . 5s

16 Repeat steps 12-15 for each sample and standard to be analyzed.

17 Ensure test tubes are labeled and place in boiling water bath for **00:20:00** .

18 Remove tubes from water bath and let cool for 00:10:00 . 10m

19 Fill plastic cuvette to line with cooled solution.

20 Place sample cuvette in spectrophotometer and record absorbance at 520 nm. Blank against the standard solution with both reagents, but no urea.

21 Use the calibration curve to determine urea concentrations of unknown solutions.
