# Supplementary material for: Colorimetric determination of urea using diacetyl monoxime with strong acids
Source: PLoS One. 2021 Nov 8;16(11):e0259760. doi: 10.1371/journal.pone.0259760 (PMC8575183; doi:10.1371/journal.pone.0259760)
Supplement: S2 File — (DOCX) [file pone.0259760.s002.docx]

Supplemental information 2. Calibration curve and urea hydrolysis data.

Table 1. Absorbances and standard deviations for urea standards using mixed color reagent (MCR) stored at 25 °C in the light and 4 °C in the dark.

|  | **25 °C MCR** | | **4 °C MCR** | |
| --- | --- | --- | --- | --- |
| Urea (mM) | Average absorbance | Standard deviation | Average absorbance | Standard deviation |
| 0 | 0 | 0 | 0 | 0 |
| 1 | 0.238 | 0.017 | 0.230 | 0.009 |
| 2 | 0.463 | 0.013 | 0.440 | 0.011 |
| 3 | 0.657 | 0.006 | 0.639 | 0.008 |
| 4 | 0.848 | 0.016 | 0.825 | 0.011 |
| 5 | 1.006 | 0.023 | 0.976 | 0.018 |

Table 2. Regression statistics for mixed color reagent (MCR) stored at 25 °C in the light and 4 °C in the dark.

| Parameter | **25 °C MCR** | **4 °C MCR** |
| --- | --- | --- |
| P | < 0.001 | < 0.001 |
| r^2^ | 0.9951 | 0.9956 |
| Standard error | 0.1466 | 0.1382 |

Table 3. Limits of detection for urea calibration curves using mixed color reagent (MCR) stored at 25 °C in the light and 4 °C in the dark. The LoD for day 4 for the 25 °C MCR curve

|  | **LoD (mM Urea)** | |
| --- | --- | --- |
| Day | 25 °C MCR | 4 °C MCR |
| 1 | 0.431 | 0.373 |
| 2 | 0.485 | 0.456 |
| 3 | 0.449 | 0.509 |
| 4 | - | 0.413 |
| 8 | 0.454 | 0.374 |
| Average | 0.455 | 0.425 |

Table 4. Concentration of nitrogen from urea in two recirculating columns over 30 days.

|  | **Urea N (mM)** | | | |
| --- | --- | --- | --- | --- |
| Day | Rep 1 | Rep 2 | Average | Standard error |
| 0 | 4.064 | 4.064 | 4.064 | 0 |
| 2 | 3.560 | 3.616 | 3.560 | 0.028 |
| 4 | 3.000 | 3.520 | 3.000 | 0.260 |
| 7 | 2.056 | 2.720 | 2.056 | 0.332 |
| 9 | 1.792 | 2.360 | 1.792 | 0.284 |
| 11 | 1.184 | 2.064 | 1.184 | 0.440 |
| 14 | 0.848 | 1.504 | 0.848 | 0.328 |
| 16 | 0.568 | 1.320 | 0.568 | 0.376 |
| 18 | 0.464 | 1.136 | 0.464 | 0.336 |
| 21 | 0.496 | 0.904 | 0.496 | 0.204 |
| 23 | 0.304 | 0.760 | 0.304 | 0.228 |
| 28 | 0.232 | 0.432 | 0.232 | 0.100 |
| 30 | 0.104 | 0.344 | 0.104 | 0.120 |

Table 5. Concentration of nitrogen from ammonium in two recirculating columns over 30 days.

|  | **Ammonium N (mM)** | | | |
| --- | --- | --- | --- | --- |
| Day | Rep 1 | Rep 2 | Average | Standard error |
| 0 | 0.009 | 0.009 | 0.009 | 0 |
| 2 | 0.390 | 0.274 | 0.332 | 0.058 |
| 4 | 0.932 | 0.498 | 0.715 | 0.217 |
| 7 | 1.719 | 1.062 | 1.391 | 0.329 |
| 9 | 2.167 | 1.546 | 1.857 | 0.311 |
| 11 | 2.600 | 1.806 | 2.203 | 0.397 |
| 14 | 2.759 | 2.224 | 2.492 | 0.268 |
| 16 | 2.903 | 2.427 | 2.665 | 0.238 |
| 18 | 3.076 | 2.744 | 2.910 | 0.166 |
| 21 | 2.816 | 2.816 | 2.816 | 0 |
| 23 | 3.236 | 2.788 | 3.012 | 0.224 |
| 28 | 3.938 | 2.860 | 3.399 | 0.539 |
| 30 | 4.189 | 2.730 | 3.460 | 0.730 |

Table 6. Concentration of total nitrogen in two recirculating columns over 30 days.

|  | **Total N (mM)** | | | |
| --- | --- | --- | --- | --- |
| Day | Rep 1 | Rep 2 | Average | Standard error |
| 0 | 4.073 | 4.073 | 4.073 | 0 |
| 2 | 3.950 | 3.890 | 3.920 | 0.030 |
| 4 | 3.932 | 4.018 | 3.975 | 0.043 |
| 7 | 3.775 | 3.782 | 3.779 | 0.004 |
| 9 | 3.959 | 3.906 | 3.933 | 0.027 |
| 11 | 3.784 | 3.870 | 3.827 | 0.043 |
| 14 | 3.607 | 3.728 | 3.668 | 0.061 |
| 16 | 3.471 | 3.747 | 3.609 | 0.138 |
| 18 | 3.540 | 3.880 | 3.710 | 0.170 |
| 21 | 3.312 | 3.720 | 3.516 | 0.204 |
| 23 | 3.540 | 3.548 | 3.544 | 0.004 |
| 28 | 4.170 | 3.292 | 3.731 | 0.439 |
| 30 | 4.293 | 3.074 | 3.684 | 0.610 |
