# Supplementary material for: Colorimetric determination of urea using diacetyl monoxime with strong acids
Source: PLoS One. 2021 Nov 8;16(11):e0259760. doi: 10.1371/journal.pone.0259760 (PMC8575183; doi:10.1371/journal.pone.0259760)
Supplement: Attachment — Submitted filename: Rebuttal to reviewer comments.docx [file pone.0259760.s003.docx]

**Rebuttal letter to original reviewer comments for ‘Colorimetric determination of urea using diacetyl monoxime with strong acids’**

4 August 2021

Dear Reviewers,

Thank you for the constructive criticism on the original version of our Lab Protocol manuscript ‘Colorimetric determination of urea using diacetyl monoxime with strong acids’. We have taken all your comments into consideration and have substantially revised the manuscript to incorporate many of your suggestions. Below you will find our point-by-point response to your suggestions in **bold**:

Reviewer #1: Dr. Langenfeld et al. has presented a work entitled as “Colorimetric determination of urea using diacetyl monoximie with strong acids”. The present method of urea estimation by complexation with diacetyl monoxime is a universally known procedure; therefore there is no novelty of the present work.

**We understand there is no novelty in the present work. That is not the purpose or intention of this lab protocol, or lab protocols in general. They are not supposed to be new methods. Rather, the intention is to simplify procedures from previous manuscripts for a well-laid out assay that can be conducted by a wide audience.**

Nevertheless this reviewer has suggested a few queries and the authors are advised to address each of the reviewers concerns in detail and resubmit the manuscript for its plausible consideration.

1. The exigency for detection/determination of urea in aqueous samples should be discussed in the introduction section. Apart from indirect enzymatic measurements the conventional methods available for urea determination (direct methods including Fearon reaction, o-pthalaldehyde, p-dimethylaminobenzaldehyde, HPLC as well as other indirect methods) and their setbacks must also be highlighted which may have instigated the authors for adapting the present protocol of urea determination.

**The new manuscript includes mention of more specific methods for urea detection. A statement on why we chose the specific method to adapt has also been included.**

2. Colorimetric determination of urea by complexation with diacetyl monoxime under acidic conditions is a well established procedure, so practically the novelty of the present work is questionable. Therefore why the present work should be considered is required to be justified.
In this consequence, the authors are advised to rewrite the introduction section with the following references in the relevant realm:
(i) {Citation}; (ii) Anal. Chem. 1955, 27, 12, 1980-1982; (iii) Clinical Chemistry, 1965, 11(2), 113-121.

**Several of these references have now been included. As mentioned prior, we know there is no novelty; that is not the purpose of this type of article.**

3. For a research paper there needs to be several experimentations validating the outcome of the analysis which is completely lacking in the present work.

**This is not a research article; it is a lab protocol. An experiment showing expected results, as outlined by PLOS guidelines, is included.**

4. The authors must study the interference effect of other biological fluids (viz. creatinine and other fluids) in the colorimetric determination of urea by the said protocol.

**These have already been studied and are cited in the manuscript. Interfering concentrations are 1000-fold less than what we would ever encounter with this protocol.**

5. The results obtained from the figures provided in the MS should be briefly discussed.

**The results are discussed near the figures.**

6. The background of the present study and the overall manuscript writing is not very appealing for the readership of the journal. Moreover, there are several typos as well as grammatical errors throughout the manuscript (viz. the term is “diacetyl monoxime” and not “Diacetyl monoxomie”. These part needs to be rectified.

**We disagree that the writing is not appealing. We feel like this method fits a PLOS Lab Protocol article perfectly. Typos have been corrected.**

7. The urea determination has been spectrophotometrically (UV-Vis absorbance) obtained and this is required to be validated with other sophisticated instrumentations like HPLC to have an in-depth perception underlying the accuracy of the present method. In addition a calibration curve should also be provided showing the results obtained for urea determination by the present method vs other reliable spectroscopic tools like HPLC with % of error in concentration evaluation.

**We do not see a reason for this validation. Data for calibration curves made from standards of reagent grade urea are shown. This is how the method is validated, from knowing the actual concentration.**

8. The overall pink colored complex formation between diacetyl monoxime and urea should be schematically provided and the chemistry underlying the complexation should also be discussed.

**A section on the chemistry of the reaction has been added.**

Reviewer #2: The authors are dealing with a topic entitled “Colorimetric determination of urea using diacetyl monoximie with strong acids” which is of limited interested to the scientific community.
A lot of points have to be improved in this manuscript:

1. The authors did not present any method used in the literature for the analytical determination of urea. Why? Any comparison is missing of the analytical assay described in this manuscript with the urea assays described in the literature.

**These comparisons have now been incorporated throughout the introduction section of the manuscript.**

2. All of the relevant analytic standard parameters such as LOD, LOQ and repeatability are missing for the characterization and validation of the analytical urea assay used in this manuscript. Why?

**The LOD, commonly given in assays for other common constituents in wastewater and hydroponic solutions, has been added. Repeatability is shown by standard error.**

3. Fig. 4: Any statistical evaluation is missing.

**Error bars and n values have been added and clarified.**

4. All of the sources of the materials and the equipment are not presented as required by PONE.

**We do not understand this concern. Instruments used in this analysis have been described.**

5. A lot of substantial references are missing.

**Additional references have been added.**

6. It should be used ml instead of mL.

**We disagree, unless this is a formatting constraint by PLOS, of which we could not locate.**

Reviewer #3: The manuscript proposes a method for the colorimetric determination of urea.

•An effort should be done in defining potential fields of application of the protocol (wastewater?, other aqueous samples? effluents from…?) to demonstrate the need of this kind of method and (in the paper) explain the benefits over other analogous protocols.

**The intentions for this protocol and its applications in wastewater and hydroponic solutions have been clarified.**

•A hypothesis about the complexation reaction and the role of the stabilising reagents (from the literature or by your own idea) should be reported to make clear to the reader possible interfering molecules that shares some properties/groups with urea.

**The reaction scheme has been described including why stabilizing agents are used. A discussion on interfering molecules and their interfering concentrations has also been added.**

•In the Introduction it is claimed that indirect methods suffer from some problems due to the interferences of some conditions in the matrix. For the proposed method is not reported any data supporting that in the conditions of the assay (pH, DMO, Thiosemicarbazide and ferric chloride concentration) different matrices (divalent ions, pH, proteins) do not affect the measurements.

**As mentioned previously, a discussion on interferences and their levels has been discussed. pH of wastewater and hydroponic solutions is not a concern for this assay.**

•In the Abstract or in the Introduction the potential fields of application of the protocol should be defined and, in the protocol itself, the absence of interferences due to the specific matrix claimed as “of interest” should be proved.

**The fields of application have now been defined. Analysis from the literature on interferences has been discussed and concluded not to be a factor in this assay.**

•Data Availability:
1.What "some restrictions will apply to data sharing" does mean about this protocol?

**This has been corrected. All data is now in the protocol or supporting information.**

•Materials and Methods:
2.the protocol reported in protocols.io should be written in extenso as M&M section with steps and concentration of reagents expressed as mM and g/L

**We do not understand this concern. In keeping with SI units, concentrations are in molarity.**

3.the pH of the solutions should be reported

**This has now been reported for the two tests conducted.**

•Results:
4.The epsilon (Extinction Coefficient) of the complex at 520 nm is not reported but only a “way to calculate” concentration and this is not rigorous.

**Epsilon has now been reported.**

5.Why the hydrolysis conditions are the ones that allow a complete hydrolysis over 30 days instead of other conditions reported in the literature (<https://doi.org/10.1002/lom3.10026>)?

**This is an example with no enzyme catalyst to start. It is simply included as a demonstration of this protocol.**

6.Nesslerization colorimetric method should be reported in more detail (at least with a reference) and the discrepancy between the initial and the final amount of total nitrogen explained.

**A reference for this method has been added and the discrepancy has been explained.**

7.The composition of at least one typical "hydroponic nutrient solution" should be reported in detail

**The composition of our hydroponic solution has been reported.**

Reviewer #4: Colorimetric determination of urea using diacetyl monoximie with strong acids
Submitted to PLOS ONE (Lab protocol)
This manuscript and laboratory protocol describes the detection of urea via the established diacetyl monoxime method using strong acids in aqueous samples. It shows linearity up to 4.6 mM urea using UV-Vis and subsequently brief application to a recirculating column system over 30 d to observed conversion of urea to ammonium. The detailed protocol is of benefit for use to the wider scientific community, given the biological and environmental importance of urea, and the variation in current methods used for urea quantification via reaction with diacetyl monoxime and detection at 520nm. As a researcher who has used this method, I fully appreciate the range of modifications published to this method, and thus the value of a standardised and detailed step-by-step protocol. The publication of a standardised method offers comparability between studies, however I have some concerns regarding the method validation (and potential caveats/interferences) and details of the application to the present example dataset.
The manuscript and lab protocol offers a standardised colorimetric method for urea, of interest to a range of disciplines, including environmental scientist, food scientists and biologists, given the biological and environmental importance of urea. However, it requires significant revisions to highlight the importance of accurate urea detection, and the importance of method standardisation, key methodological details (e.g. limit of detection, reagent and chromophore stability), suitability of this approach, including potential caveats/interferences with this method and details regarding the example dataset provided. There are relatively recent detailed publications, including validation for other aqueous matrices (as cited within as ref 4), thus such a protocol must build upon this. I detail both major and minor comments below.

Major comments:
1. Throughout the text, and within the title and uploaded protocol, monoxime is spelt as “monoximie” and needs to be corrected.

**This has been corrected.**

2. It is touched upon within the abstract the importance of urea as a waste product of mammalian N metabolism, and in agriculture, however, the introduction of overly brief with respect the importance of urea (i.e. most common fertiliser worldwide; proportion of N in ruminant urine, importance in waste water) and the subsequent potential environmental impacts. Highlighting the importance of accurately detecting urea in environmental matrices within the introduction would add to the power of the manuscript.

**The introduction has been expanded to include the importance of analyzing urea in environmental samples.**

3. Furthermore, further details of the diacetyl monoxime method would be of benefit within the introduction (e.g. previous application, key reaction steps, and variations on the method (e.g. automated and manual, difference stabilisation of the chromophore)). Given this would represent a “go-to” methodology for this method, including these fundamental details would greatly benefit this manuscript, and future readers to understand suitable applications.

**The development of the method and its variations have now been included.**

4. It is touched upon within the introduction that high protein content may reduce accuracy of urea and urea derivatives via this approach. This is the only area of the manuscript that you note that the method experiences varying interferences from urea derivatives (e.g. compounds in ruminant urine and non-protein feed). Given this is then applied in a recirculating system for urea removal, in a real world setting, this would be a complex matrix potentially including compounds which also produce a chromophore which is detected at 520 nm under the reagent conditions, or result in interference in the reaction, it is vital to highlight this in the introduction, accurately describing what is detected (i.e. urea and urea derivatives) and potential interferences. These caveats are essential for the publication of such a laboratory protocol, to allow a reader to fully evaluate the suitability of this approach, including references to original literature to refer to (however old) for further details (e.g. Archibald, Journal of Biological Chemistry 1944 and 1945, and more recently Reay et al., 2019, SBB).

**These references have been included. A discussion on interferences and why they are not an issue in this version of the assay have also been added.**

5. In the uploaded protocol, there is no reference to the stability of prepared reagents (i.e. do these need to be made daily/ how long they are stable for). Common automated methods (e.g. available from Skalar) include such details, and are vitally important for such colorimetric methods when conducted both manually (as within this manuscript) or automated. For example, colour regent stability has been shown to decrease within 3 days of preparation (Reay et al., 2019 SBB), thus including such details in a standardised automated method would ensure inter-lab comparability and add quality control to the protocol.

**This is now discussed in the introduction and is now a substantial part of the example data set provided for this protocol. We appreciate this suggestion!**

6. In the abstract (line 37), introduction (L50), and protocol, it states absorbance is linear to 4.6 mM, however on L60, it suggested it was linear to 5 mM. Given this is almost 10% of the range of the method, please clarify the range of linearity. Furthermore, as you give the upper range of this method, including a limit of detection within this protocol would avoid potential unsuitable application to aqueous matrices with low urea concentrations (e.g. sea water – as referenced within the manuscript (ref 4)).

**The range, both upper and lower, has been clarified.**

7. The expected result use a recirculating column system I assume is relevant for waste water treatment and removal of urea. It would be useful to expand on this, so the reader can understand the environmental relevance of the example dataset you have presented. This includes the role of the column in urea hydrolysis, given line 72-73 states “urea is hydrolysed under acidic conditions in the column” to highlight this would occur in the column, but not in the strong acidic conditions of the colorimetric method.

**The use of this system and its design have now been clarified in the manuscript.**

8. Line 79-80 states this method has also been tested in a background of hydroponic nutrient solution. Can you expand on what this was, as it will be useful for the reader to know what the matrix was, when no interferences were observed? This is particularly important as a number of interferences have been observed with the diacetyl monoxime method (see comment 3).

**The composition of the hydroponic nutrient solution has now been clarified.**

Minor comments:
1. In title, change “monoximie” to “monoxime”

**Done.**

2. In key words, change “diacatyl” to “diacetyl”

**Done.**

3. Line 62-64: Please clarify where these numbers in the calculations come from.

**This section has been removed and replaced with epsilon.**

4. While the main focus of this manuscript is to report the suitability of the protocol for urea, details of other methods (e.g. Nesslerization colorimetric method L71) used for data presented within the manuscript must also be included (e.g. a reference for this, and a brief description). This can be with the caveat this are established methods, and thus are not included within the lab protocol, but will be useful for a reader to fully understand the example dataset.

**A reference for the nesslerization method has now been included in the manuscript.**

5. Line 72 and 73, and Figure 4 shows declining total N in the recirculating system after ca. 17 days, and ammonium concentration almost plateaus, while urea concentration continues to decline. I presume it is a closed system from Figure 3- where are the N losses occurring? Also in Figure 4, I cannot see any error bars? The text suggests n=4 – are there error bars (or are they so small they are not visible – if so please clarify in the caption). Furthermore, does the y axis account for differing different moles N l-1 for urea (2N) vs. ammonium (1N)? It is not clear- for every 1 urea molecule hydrolysed = 2 NH4+, but increases in ammonium relative decline to urea is not double?

**A clarification on N losses has now been included in the manuscript. Error bars have been added and the n value for the figure has been clarified. A clarification on how the N displayed was measured has also been added.**

Sincerely,

Noah J. Langenfeld

Lauren E. Payne

Bruce Bugbee
