# Supplementary material for: Colorimetric determination of urea using diacetyl monoxime with strong acids
Source: PLoS One. 2021 Nov 8;16(11):e0259760. doi: 10.1371/journal.pone.0259760 (PMC8575183; doi:10.1371/journal.pone.0259760)
Supplement: Attachment — Submitted filename: Rebuttal to reviewer comments 6 October 2021.docx [file pone.0259760.s004.docx]

**Rebuttal letter to original reviewer comments for ‘Colorimetric determination of urea using diacetyl monoxime with strong acids’**

6 October 2021

Dear Reviewers,

Thank you for the constructive criticism on the original version of our Lab Protocol manuscript ‘Colorimetric determination of urea using diacetyl monoxime with strong acids’. We have taken all your comments into consideration and have substantially revised the manuscript to incorporate many of your suggestions. Below you will find our point-by-point response to your suggestions in **bold**:

Reviewer #2:

The protocol, that is not included in M&M section despite my suggestion in the first revision, but only available as supplementary material, is written in an unclear/uncorrect form: **This was done because we were following the template for a lab protocol laid out by PLOS One. Lab protocols are meant to have the protocol included only as supplementary information and a link placed in the materials and methods section with a DOI. No change has been made because of this reason.**

• point nr 2: Add 80 µl phosphoric acid. Unclear: Which is the concentration of the Phosphoric Acid? **The concentration has been added to the protocol.**

• point nr 3: Prepare 18 Molarity (M) sulfuric acid by diluting 65.25 mL concentrated sulfuric acid up to 250 mL with deionized water. This is uncorrect: 18 M Sulfuric Acid IS concentrated Sulfuric acid: Concentrated Sulfuric Acid 98% (H2SO4) MW: 98.073 Density: 1.84 Approx. Strength: 96% Molarity(M): 18 Volume (mL) required to make 1000 mL of 1M solution: 55.

**You are correct, this was a mistake on our part and the correct concentration has now been included in the protocol.**

• The correct spelling of [mM] is Millimolar, not Milimolar. **This was a default setting on protocols.io. We have corrected the spelling.**

• Purity of reagents (thiosemicarbazide, diacetyl monoxime, phosphoric acid, ferric chloride) is not reported. **Purity has now been reported for all reagents.**

Sincerely,

Noah J. Langenfeld, Lauren E. Payne, and Bruce Bugbee
